# Association Between *MTHFR* Polymorphisms and the Risk of Essential Hypertension: An Updated Meta-analysis

**DOI:** 10.3389/fgene.2021.698590

**Published:** 2021-11-26

**Authors:** Hao Meng, Shaoyan Huang, Yali Yang, Xiaofeng He, Liping Fei, Yuping Xing

**Affiliations:** ^1^ Department of Cardiovascular Medicine, Heping Hospital Affiliated to Changzhi Medical College, Changzhi, China; ^2^ Department of Endocrinology, Shaogauan First People’s Hospital, Shaoguan, China; ^3^ Department of science and education, Heping Hospital Affiliated to Changzhi Medical College, Changzhi, China; ^4^ Department of Cardiovascular Medicine, Heji Hospital Affiliated to Changzhi Medical College, Changzhi, China; ^5^ Neurology Department, Heping Hospital Affiliated to Changzhi Medical College, Changzhi, China

**Keywords:** MTHFR, Rs1801133, Rs1801131, Essential hypertension, FPRP, BFDP, Venice criteria

## Abstract

Background: Since the 1990s, there have been a lot of research on single-nucleotide polymorphism (SNP) and different diseases, including many studies on 5,10-methylenetetrahydrofolate reductase (*MTHFR*) polymorphism and essential hypertension (EH). Nevertheless, their conclusions were controversial. So far, six previous meta-analyses discussed the internal relationship between the *MTHFR* polymorphism and EH, respectively. However, they did not evaluate the credibility of the positive associations. To build on previous meta-analyses, we updated the literature by including previously included papers as well as nine new articles, improved the inclusion criteria by also considering the quality of the papers, and applied new statistical techniques to assess the observed associations. Objectives: This study aims to explore the degree of risk correlation between two *MTHFR* polymorphisms and EH. Methods: PubMed, EMBASE, the Cochrane Library, CNKI, and Wan Fang electronic databases were searched to identify relevant studies. We evaluated the relation between the *MTHFR C677T* (rs1801133) and *A1298C* (rs1801131) polymorphisms and EH by calculating the odds ratios (OR) as well as 95% confidence intervals (CI). Here we used subgroup analysis, sensitivity analysis, cumulative meta-analysis, assessment of publication bias, meta-regression meta, False-positive report probability (FPRP), Bayesian false discovery probability (BFDP), and Venice criterion. Results: Overall, harboring the variant of *MTHFR C677T* was associated with an increased risk of EH in the overall populations, East Asians, Southeast Asians, South Asians, Caucasians/Europeans, and Africans. After the sensitivity analysis, positive results were found only in the overall population (TT *vs.* CC: OR = 1.14, 95% CI: 1.00–1.30, *P*_h_ = 0.032, *I* ^2^ = 39.8%; TT + TC *vs.* CC: OR = 1.15, 95% CI: 1.01–1.29, *P*_h_ = 0.040, *I* ^2^ = 38.1%; T *vs.* C: OR = 1.14, 95% CI: 1.04–1.25, *P*_h_ = 0.005, *I* ^2^ = 50.2%) and Asian population (TC *vs.* CC: OR = 1.14, 95% CI: 1.01–1.28, *P*_h_ = 0.265, *I* ^2^ = 16.8%; TT + TC *vs.* CC: OR = 1.17, 95% CI: 1.04–1.30, *P*_h_ = 0.105, *I* ^2^ = 32.9%; T *vs.* C: OR = 1.10, 95% CI: 1.02–1.19, *P*_h_ = 0.018, *I* ^2^ = 48.6%). However, after further statistical assessment by FPRP, BFDP, and Venice criteria, the positive associations reported here could be deemed to be false-positives and present only weak evidence for a causal relationship. In addition, when we performed pooled analysis and sensitivity analysis on *MTHFR A1298C*; all the results were negative. Conclusion: The positive relationships between *MTHFR C677T* and *A1298C* polymorphisms with the susceptibility to present with hypertension were not robust enough to withstand statistical interrogation by FPRP, BFDP, and Venice criteria. Therefore, these SNPs are probably not important in EH etiology.

## Introduction

Essential hypertension (EH) is a common disease in the world and a threat to the health of people. If blood pressure is not well controlled, it can lead to serious consequences, such as coronary heart disease, enlarged heart, heart failure, cerebral hemorrhage, optic papillary edema, and renal insufficiency. At the same time, chronic high blood pressure could lead to clinical symptoms such as dizziness, headache, chest pain, tinnitus, vomiting, palpitation, and blurred vision (O'Donnell et al., 1998). EH is also called primary hypertension because its etiology is not clear, and there is, so far, no complete understanding thereof (Bähr et al., 2003). It is the result of the interaction of various environmental factors and genetic factors. The former mainly includes diet, smoking, mental stress, and lifestyle. The latter includes contributors such as obesity, family history, and genetic variations or polymorphisms (Singh et al., 2016). In recent years, many genes related to hypertension have been reported, and relevant evidence suggested that genetic factors accounted for about 25–65% of the proportion in those with hypertension (Krzesinski and Saint-Remy, 2012). Therefore, genetic factors are critical to explore as a possible internal cause of this disease. In Europe, Newton-Cheh *et al*. conducted a genome-wide association study (GWAS) which used blood pressure as a continuous trait (Newton-Cheh et al., 2009). Through a two-stage meta-analysis, three loci associated with systolic blood pressure (*MTHFR*, *CYP17A1*, and *PLCD3*) and five diastolic blood pressure loci (*FGF5*, *C10orf107*, *SH2B3*, *CYP1A2*, and *ZNF652*) were identified at the genome-wide level. This is of great significance for the genetic research of EH (Rafiq et al., 2010).

Studies have shown that hyperhomocysteinemia (hHcy) is an important risk factor for hypertension. 5,10-Methylenetetrahydrofolate reductase (*MTHFR*) plays an important role in folate metabolism–methionine cycle. Together with methionine synthase reductase (MTRR), it maintains the normal metabolism of folate and participates in the maintenance of normal homocysteine (Hcy) levels in the body. Specific *MTHFR* gene mutations can lead to a decrease in the activity of key enzymes and disorders in folate metabolism, which increased the demand for folate to maintain Hcy methylation to methionine (Met) and ultimately lead to the increase of Hcy level and hHcy (Ren et al., 2018). The human *MTHFR* gene is located in autosomal 1p36.3. There are a variety of mutation types and multiple mutation sites in the *MTHFR* gene, of which *C677T* (rs1801133) and *A1298C* (rs1801131) are two common gene sites. The 677C-T gene mutation is located in exon 4 of the catalytic activity of the N-terminal region, where cytosine is replaced by thymine, and the corresponding protein is changed from alanine (Ala) to valine (Val). The *1298A-C* mutation is located in the C-terminal regulatory region of exon 7, where adenosine mutates to cytosine, causing the glutamate (Glu) encoding to be replaced by alanine (Ala) (Weisberg et al., 2001; Chen et al., 2009; Nursal et al., 2018). Globally, the ethnic and geographic distribution of these two loci are significantly different. In 2010, approximately 1.39 billion people suffered from EH, especially in low- and middle-income countries (Kearney et al., 2005; Yang and Gu, 2016; Gheorghe et al., 2018; Mills et al., 2020). Therefore, EH was a major risk factor contributing to the global burden of disease (Falaschetti et al., 2014). Although many studies have elucidated the pathogenesis, the associations between the *MTHFR* SNPs and blood pressure were unclear (Doris, 2002; Staessen et al., 2003). Studies have shown that EH was related to the inheritance of a variety of susceptibility genes.

Researchers have explored the susceptibility conveyed *via* the *MTHFR* gene polymorphisms to develop EH. There are many published meta-analyses, but the conclusions were controversial (Qian et al., 2007; Niu et al., 2012; Yang B. et al., 2014; Yang K.-M. et al., 2014; Wu et al., 2014; Fu et al., 2019). Researchers have also continued to conduct various population-based studies wherein they explored the relationship between specific *MTHFR* gene variations and EH, but the results were different (Nishio et al., 1996; Sohda et al., 1997; Nakata et al., 1998; Gao et al., 1999; Powers et al., 1999; Zhan et al., 2000a; Zhan et al., 2000b; Kobashi et al., 2000; Li et al., 2000; Rajkovic et al., 2000; Zusterzeel et al., 2000; Benes et al., 2001; Kahleová et al., 2002; Wang et al., 2002; Rodríguez-Esparragón et al., 2003; Sun et al., 2003; Frederiksen et al., 2004; Heux et al., 2004; Liu et al., 2004; Yilmaz et al., 2004; Cesari et al., 2005; Liu et al., 2005; Tylicki et al., 2005; Demir et al., 2006; Hu et al., 2006; Kalita et al., 2006; Li and Huang, 2006; Lwin et al., 2006; Deng, 2007; Hu et al., 2007; Hui et al., 2007; Marinho et al., 2007; Markan et al., 2007; Nagy et al., 2007; Tang et al., 2007; Xing and Hua, 2007; Canto et al., 2008; Fridman et al., 2008; Ilhan et al., 2008; Lin et al., 2008; Luo et al., 2008; Soares et al., 2008; Cai and Gong, 2009; Deshmukh et al., 2009; Fakhrzadeh et al., 2009; Ng et al., 2009; Wang et al., 2010a; Wang et al., 2010b; Yu, 2010; Liu et al., 2011a; Liu et al., 2011b; Demirel et al., 2011; Jin et al., 2011; Ma and Yang, 2011; Mendilcioglu et al., 2011; Su et al., 2011; Alghasham et al., 2012; Cao, 2012; Fowdar et al., 2012; Yin et al., 2012; Zhang et al., 2012; Bayramoglu et al., 2013; Fridman et al., 2013; Yang et al., 2013; Yao et al., 2013; Cai et al., 2014; Husemoen et al., 2014; Vazquez-Alaniz et al., 2014; Bayramoglu et al., 2015; Nassereddine et al., 2015; Pérez-Razo et al., 2015; Wang et al., 2015; Wei et al., 2015; Wen et al., 2015; Amrani-Midoun et al., 2016; Fan et al., 2016; Ghogomu et al., 2016; Wu and Xu, 2016; Dwivedi and Sinha, 2017; Rios et al., 2017; Zhang et al., 2017; Zhao et al., 2017; Arina et al., 2019; Liu et al., 2019; Nong et al., 2019; Wu et al., 2019; Zhao et al., 2019; Cai et al., 2020; Candrasatria et al., 2020)—for example, three studies (Nakata et al., 1998; Wen et al., 2015; Fan et al., 2016) indicated that *C677T* was a risk gene locus for EH. However, other studies have found no correlation between them (Rodríguez-Esparragón et al., 2003; Amrani-Midoun et al., 2016). Similarly, two studies (Kahleová et al., 2002; Alghasham et al., 2012) reported the correlation between *A1298C* and EH, but others (Ng et al., 2009; Wei et al., 2015) believed that *A1298C* did not play a role in the pathogenesis of EH. Because of the controversies in the field, several meta-analyses incorporating relevant studies were conducted, but there were still some shortcomings in the methods of these systematic summaries with statistical analysis of the available literature. Firstly, there were obvious errors in incorporating certain studies that included hypertension experienced in pregnancy within two of the existing meta-analyses. The latter is problematic because extrapolating the results of these meta-analyses to apparently healthy individuals of the general population will not be permissible—for example, 11 studies on pregnancy-induced hypertension were mistakenly included in a meta-analysis (Fu et al., 2019), as described below (Powers et al., 1999; Kobashi et al., 2000; Li et al., 2000; Rajkovic et al., 2000; Zusterzeel et al., 2000; Yilmaz et al., 2004; Demir et al., 2006; Nagy et al., 2007; Canto et al., 2008; Vazquez-Alaniz et al., 2014; Rios et al., 2017). Similarly, authors of another meta-analysis (Yang B. et al., 2014) also mistakenly included four studies on hypertensive disorders of pregnancy (Sohda et al., 1997; Yu, 2010; Mendilcioglu et al., 2011; Su et al., 2011). Secondly, several meta-analyses authors did not carry out literature quality assessment (Qian et al., 2007; Niu et al., 2012). Finally, they did not assess the reliability of the statistical association and the levels of cumulative epidemiological evidence (Qian et al., 2007; Niu et al., 2012; Yang B. et al., 2014; Yang K.-M. et al., 2014; Wu et al., 2014; Fu et al., 2019). Therefore, we addressed the shortcomings of the previous meta-analyses investigating the relationships between the common *MTHFR* SNPs at loci *677* and *1298* with hypertension. Additionally, we included evidence from new case–control studies that were not included previously but increased the sample size and the reliability of our findings.

## Materials and Methods

### Search Strategy

Databases, including PubMed, EMBASE, the Cochrane Library, CNKI, and Wan Fang databases, were searched for evidence between the carrier status of the *MTHFR* gene variants and susceptibility to EH. The retrieval strategy was as follows: (*MTHFR C677T* OR rs1801133 OR Ala222Val) AND (*MTHFR A1298C* OR rs1801131 OR Glu429Ala) AND (polymorphism OR mutation OR variant OR genotype) AND (essential hypertension OR hypertension OR EH OR blood pressure). Two researchers who were familiar with the literature search process and meta-analysis methods conducted a literature search to identify all possible original studies. The search deadline was December 2020.

### Selection Criteria

The inclusion criteria were as follows: (1) case–control study, (2) complete study of genotype data, and (3) study exploring the relationship between *MTHFR* polymorphisms and EH.

The exclusion criteria were as follows: (1) unrelated diseases or other diseases associated with hypertension or secondary hypertension, (2) incomplete study of genotype data and genotype frequency, (3) duplicate study, and (4) letters, reviews, animal experiments, other analysis.

### Data Extraction

According to the abovementioned inclusion and exclusion criteria, two researchers searched independently the abovementioned databases and finally checked them to ensure a comprehensive search and information extraction of all relevant studies. If there was a difference in opinion, it would be discussed with a third researcher. The extraction will be based on the following information: last name of the first author, publication year, country, geographical location, ethnicity of the study subjects, source of the case group, source of the control group, matching, diagnostic criteria for EH, genotype data in cases and controls, sample size, genotype examination, and adjustments.

### Quality Assessment

All studies were independently evaluated and verified by two investigators. According to previously published articles (Thakkinstian et al., 2011; Xue et al., 2014) together with the characteristics of this study, a quality assessment table was designed. The maximum score a study could achieve based on the quality assessment criteria was 20. We regarded studies achieving a score of 12 and higher as good-quality research. Those scoring below 12 were not eligible for inclusion based on the low quality thereof. The specific evaluation criteria are shown in Table 1.

**TABLE 1:** Scale for the quality assessment of molecular association studies of essential hypertension.

Criterion	Score
Source of case	
Selected from population	2
Selected from hospital	1
Not described	0
Source of control	
Population-based	3
Blood donors or volunteers	2
Hospital-based	1
Not described	0
Ascertainment of essential hypertension	
International diagnostic criteria	2
Regional diagnostic criteria	1
Not described	0
Ascertainment of control	
Controls were tested to screen out EH	2
Controls were subjects who did not report EH, no objective testing	1
Not described	0
Matching	
Controls matched with cases by age and sex	2
Controls matched with cases only by age or sex	1
Not matched or not described	0
Genotyping examination	
Genotyping done blindly and quality control	2
Only genotyping done blindly or quality control	1
Unblinded and without quality control	0
HWE	
HWE in the control group	2
Hardy–Weinberg disequilibrium in the control group	1
Not described	0
Association assessment	
Assess association between genotypes and EH with appropriate statistics and adjustment for confounders	2
Assess association between genotypes and EH with appropriate statistics without adjustment for confounders	1
Inappropriate statistics used	0
Total sample size	
>1,000	3
500–1,000	2
200–500	1
<200	0

HWE, Hardy–Weinberg equilibrium; EH, essential hypertension.

### Credibility Analysis

The reliability of the statistically significant association was evaluated by false-positive reporting probability (FPRP), Bayesian false discovery probability (BFDP) test, and Venice criterion (Ioannidis et al., 2008). When FPRP <0.2 and BFDP <0.8, based on a predetermined prior probability of 0.001, the results of the genetic association were valid, indicating that the association might be true (Wakefield, 2007). The Venice criterion evaluated the credibility of cumulative results in terms of validity of evidence, reproducibility of studies, and bias control. The evaluation indicators were as follows: (1) evidence validity (*n*: genotype sample size): A: *n* ≥ 1,000; B: 100 ≤ *n* < 1,000; C: *n* < 100; (2) reproducibility: A: *I* ^2^ <25%; B: *I* ^2^ >25% and *I* ^2^ < 50%; C: *I* ^2^ >50% or higher; and (3) bias control: A: no bias; B: no obvious bias but data is missing; C: obvious bias. A, B, and C represent strong, moderate, and weak cumulative epidemiological evidence, respectively (Wakefield, 2007; Ioannidis et al., 2008).

### Statistical Analysis

Chi-square goodness-of-fit test was used to perform Hardy–Weinberg equilibrium (HWE) on the included control group and to select the studies conforming to HWE. We calculated the combined odds ratio (OR) and 95% confidence intervals (CI) to determine the association between *MTHFR* gene polymorphisms and EH. Evaluation of heterogeneity was by Cochran *Q* test and *I* ^2^ test. When *P* >0.10 and/or *I* ^2^ ≤50%, a fixed-effect model was selected to combine the effect sizes. When *P* ≤0.10 and/or *I* ^2^ >50%, a random-effects model was selected (Higgins et al., 2003). Allelic model, dominant model, recessive model, heterozygote model, and homozygote genetic model were used for the evaluation (Fu et al., 2019). We could identify the sources of heterogeneity in the following ways: (1) meta-regression was performed on the factors that might lead to heterogeneity in the study itself; (2) according to ethnicity, geographical distribution, and genetic testing quality, a subgroup analysis was conducted; and (3) we conducted a sensitivity analysis by excluding individual studies one by one and judged the publication bias by Begg’s funnel plot and Egger test. If there was publication bias, we would use the trim-and-fill method to correct it (Zhang et al., 2018). Cumulative meta-analysis of included literature was performed to dynamically observe the results (Lau et al., 1992). STATA12.0 software was used for statistical analysis, and *P* <0.05 was considered to be statistically significant.

## Results

### Description of the Included Studies

**FIGURE 1:**
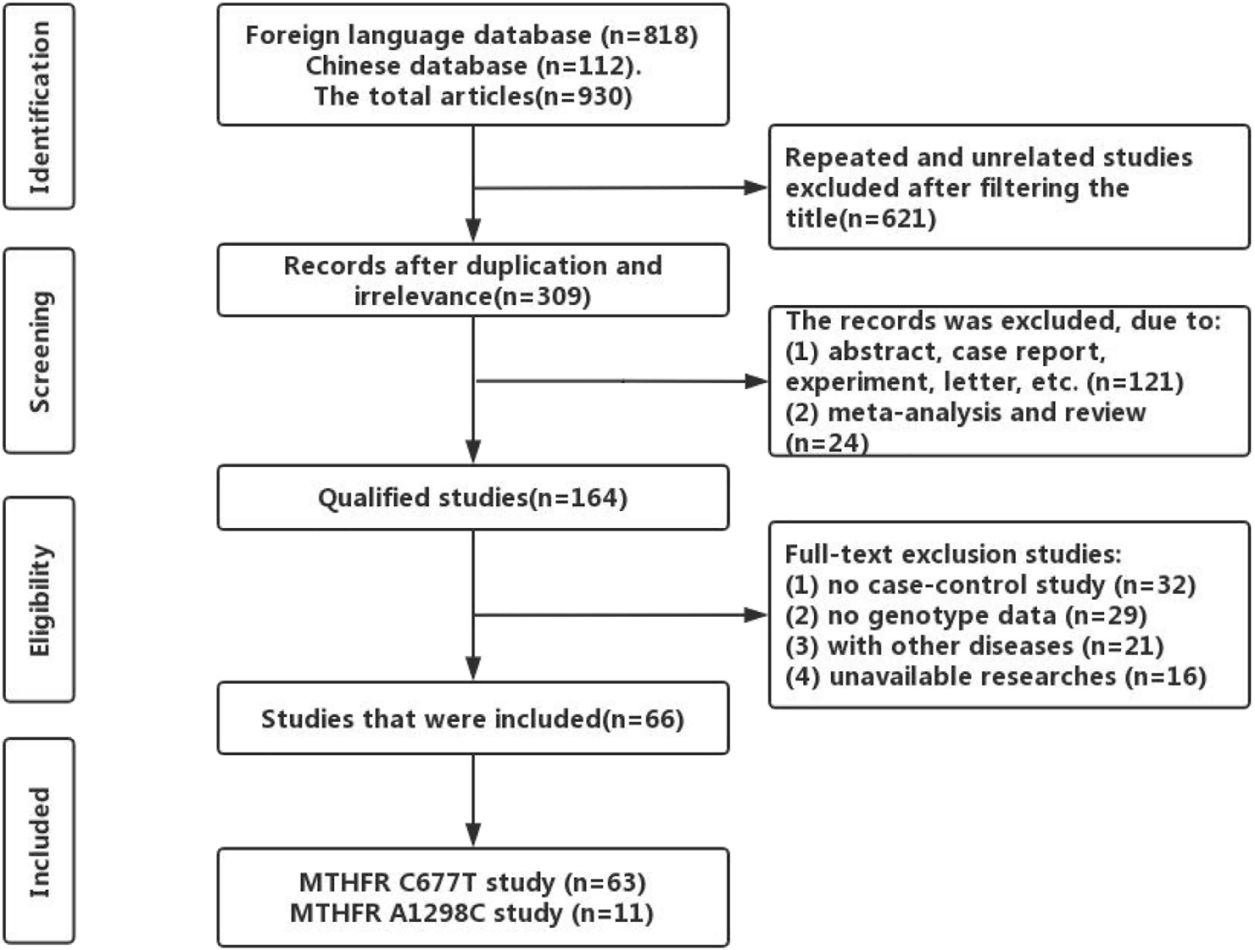
Flow chart of literature selection for the current meta-analysis.

Based on STREGA and PRISMA (Swartz, 2011), we carried out this research. According to the retrieval method, we searched PubMed, Embase, the Cochrane Library, CNKI, and Wan Fang electronic databases and obtained 930 articles. According to strict inclusion and exclusion criteria, 66 original literatures were finally included in this study, including 63 articles on *C677T* polymorphism and 11 articles on *A1298C* polymorphism (see Figure 1 for the detailed flow chart of the included literature).

### Basic Characteristics and Quality Evaluation of Literature

A total of 66 articles were included, of which 63 were about *MTHFR C677T* and EH and 11 were about *MTHFR A1298C*. The results of the quality assessment were 25 high-quality studies and 41 low-quality studies. There were 58 studies in the control group that complied with HWE. The specific features of the included literature are shown in Table 2. The detailed genotype distribution is shown in Table 3 and Table 4.

**TABLE 2:** General characteristics of the included studies and literature quality scores.

First author/Year	Country	Geographic region	Ethnicity	Source of cases	Source of controls	Matching	Sex	Quality scores
*C677T*								
Nishio [22] 1996	Japan	EA	A	HB	HB	No	M	7
Nakata [24] 1998	Japan	EA	A	HB	HB	Age, sex	–	10
Gao [25] 1999	China	EA	EA	PB	PB	No	M/W	13
Zhan [30] 2000	China	EA	A	PB	PB	No	M/W	13
Zhan [31] 2000	China	EA	EA	PB	PB	No	M/W	13
Benes [33] 2001	Czech Republic	E	C	HB	PB	No	M/W	8
Kahleova [34] 2002	Czech Republic	E	C	HB	Volunteers	No	M/W	9
Wang [35] 2002	China	EA	A	HB	HB	No	M/W	7
Rodríguez-Esparragón [36] 2003	Spain	E	C	PB	PB	Age, sex	M/W	14
Sun [37] 2003	China	EA	A	HB	HB	No	M/W	8
Heux [39] 2004	Australia	O	C	NR	NR	Age, sex	M/W	11
Liu [41] 2004	China	EA	A	PB	PB	No	M/W	12
Cesari [42] 2005	Italy	E	C	NR	NR	No	M/W	8
Liu [43] 2005	China	EA	EA	PB	PB	No	M/W	12
Tylicki [44] 2005	Austria/Poland	O	C	HB	HB	Age, sex	–	12
Hu [46] 2006	China	EA	A	PB	PB	Age, sex	M/W	15
Li [48] 2006	China	EA	A	HB	HB	No	M/W	7
Lwin [49] 2006	Japan	EA	A	PB	PB	No	M	11
Hu [50] 2007	China	EA	EA	PB	PB	Age, sex	M/W	15
Markan [52] 2007	India	SA	A	HB	HB	Age, sex	M/W	12
Tang [54] 2007	China	EA	A	HB	HB	No	M/W	10
Xing [55] 2007	China	EA	A	HB	PB	Age	M/W	15
Hui [56] 2007	Japan	EA	A	PB	PB	No	M/W	13
Deng [57] 2007	China	EA	A	HB	PB	Age, sex	M/W	14
Fridman [59] 2008	Argentina	Af	M	HB	HB	No	M/W	10
Ilhan [60] 2008	Turkey	WA	C	HB	HB	Age	M/W	9
Lin [61] 2008	China	EA	A	HB	HB	Age, sex	M/W	11
Luo [62] 2008	China	EA	A	HB	HB	No	M/W	10
Soares [63] 2008	Brazil	SAm	M	NR	Volunteers	Age, sex	M/W	11
Deshmukh [65] 2009	United States	NAm	C	PB	Volunteers	No	M/W	9
Fakhrzadeh [66] 2009	Iran	WA	C	PB	PB	No	M/W	11
Ng [67] 2009	Australia	E	C	PB	PB	No	M/W	10
Liu [69] 2011	China	EA	A	HB	HB	No	M/W	9
Liu H [70] 2011	China	EA	A	HB	HB	No	M/W	10
Jin [72] 2011	China	EA	A	HB	HB	Age, sex	M/W	12
Ma [73] 2011	China	EA	A	HB	HB	No	M/W	8
Cao [75] 2012	China	EA	A	HB	HB	Age, sex	M/W	12
Fowdar [76] 2012	Australia	O	C	PB	PB	Age, sex	M/W	15
Yin [77] 2012	China	EA	A	PB	PB	Age, sex	M/W	16
Zhang [78] 2012	China	EA	A	HB	PB	No	M/W	12
Alghasham [79] 2012	Saudi Arabia	WA	C	HB	HB	No	M/W	7
Bayramoglu [80] 2013	Turkey	WA	C	HB	HB	No	M/W	7
Fridman [81] 2013	Argentina	Af	C	HB	HB	No	M/W	10
Yao [82] 2013	China	EA	A	HB	HB	Age, sex	M/W	10
Yang [83] 2013	China	EA	A	HB	HB	No	M/W	11
Cai [84] 2014	China	EA	EA	PB	PB	No	M/W	14
Bayramoglu [87] 2015	Turkey	WA	C	HB	HB	No	M/W	7
Nassereddine [88] 2015	Morocco	Af	C	HB	HB	Age, sex	M/W	12
Wei [90] 2015	Malaysia	SEA	A	HB	HB	No	M/W	11
Wen [91] 2015	China	EA	A	NR	NR	No	M/W	8
Pérez-Razo [92] 2015	Mexico	NAm	M	HB	Blood donors	No	M/W	13
				PB	PB	Age, sex	M/W	15
Amrani-Midoun [93] 2016	Argentina	Af	C	HB	Blood donors	No	M/W	9
Fan [94] 2016	China	EA	A	HB	HB	No	M/W	11
Dwivedi [95] 2017	India	SA	A	HB	HB	No	–	6
Wu [98] 2016	China	EA	A	HB	HB	No	M/W	7
Ghogomu [99] 2016	Cameroun	Af	Af	HB	PB	No	M/W	10
Zhang [100] 2017	China	EA	A	HB	HB	No	M/W	8
Zhao [101] 2017	China	EA	A	HB	HB	No	M/W	11
Liu [102] 2019	China	EA	A	PB	PB	No	M/W	16
Nong [103] 2019	China	EA	A	PB	PB	No	M/W	11
Wu [104] 2019	China	EA	A	HB	HB	No	M/W	9
Zhao [105] 2019	China	EA	A	HB	PB	No	M/W	10
Candrasatria [106] 2020	Indonesia	SEA	A	PB	PB	No	M/W	13
*A1298C*								
Kahleova [34] 2002	Czech Republic	E	C	HB	Volunteers	No	M/W	9
Tylicki [44] 2005	Austria/Poland	O	C	HB	HB	Age, sex	–	12
Markan [52] 2007	India	SA	A	HB	HB	Age, sex	M/W	11
Ng [67] 2009	Australia	E	C	PB	PB	No	M/W	10
Wang [107] 2010	China	EA	A	PB	PB	No	M/W	13
Wang [108] 2010	China	EA	A	PB	PB	No	M/W	15
Demirel [109] 2011	Turkey	WA	C	PB	PB	No	M/W	8
Fowdar [76] 2012	Australia	O	C	PB	PB	Age, sex	M/W	15
Alghasham [79] 2012	Saudi Arabia	WA	C	HB	HB	No	M/W	7
Wei [90] 2015	Malaysia	SEA	A	HB	HB	No	M/W	11
Liu [102] 2019	China	EA	A	PB	PB	No	M/W	16

HB, hospital-based study; PB, population-based study; EA, East Asia; SA, South Asia; WA, Western Asia; SEA, Southeast Asia; O, Oceania; E, Europe; Af, Africa; SAm, South America; NAm, North America; A, Asian; C, Caucasian; M, mixed; M/W, men/women.

**TABLE 3:** The genotype distribution and HWE of *MTHFR C677T* polymorphism in this meta-analysis. The bold value is C/T T/T A/C C/C genotype data is incomplete, and HWE evaluation cannot be performed.

First author/Year	Number of samples	Genotype of cases			Genotype of controls			HWE	
	Cases/controls	C/C	C/T	T/T	C/C	C/T	T/T	Chi	*p*
Nishio [22] 1996	47/82	16	26	5	29	44	9	1.631	0.2015
Nakata [24] 1998	173/184	63	91	19	65	83	36	1.031	0.3100
Gao [25] 1999	127/170	44	68	15	62	84	24	0.275	0.6001
Zhan [30] 2000	127/170	44	68	15	62	84	24	0.275	0.6001
Zhan [31] 2000	127/170	44	68	15	62	84	24	0.275	0.6001
Benes [33] 2001	193/209	73	93	27	86	106	17	4.005	0.0454
Kahleova [34] 2002	164/173	82	55	27	86	69	18	0.553	0.4570
Wang [35] 2002	105/46	17	51	37	14	23	9	0.007	0.9354
Rodríguez-Esparragón [36] 2003	232/215	83	115	34	95	100	20	0.751	0.3861
Sun [37] 2003	55/46	6	22	27	14	23	9	0.007	0.9354
Heux [39] 2004	247/249	87	125	35	105	119	25	1.080	0.2988
Liu [41] 2004	100/100	29	45	26	31	50	19	0.021	0.8838
Cesari [42] 2005	100/101	36	39	25	32	49	19	0.001	0.9748
Liu [43] 2005	100/100	29	45	26	31	50	19	0.021	0.8838
Tylicki [44] 2005	90/90	40	39	11	42	38	10	0.100	0.7517
Hu [46] 2006	157/115	75	55	33	61	42	12	1.330	0.2488
Li [48] 2006	26/30	18	6	2	21	7	2	1.462	0.2266
Lwin [49] 2006	116/219	39	58	19	64	117	38	1.537	0.2151
Hu [50] 2007	110/115	55	39	16	61	42	12	1.330	0.2488
Markan [52] 2007	153/133	105	40	8	105	28	0	1.841	0.1749
Tang [54] 2007	252/195	139	93	20	138	51	6	0.232	0.6298
Xing [55] 2007	686/509	202	184	300	182	105	222	174.111	0
Hui [56] 2007	261/271	83	129	49	104	123	44	0.560	0.4542
Deng [57] 2007	151/138	108	35	8	91	40	7	0.863	0.3529
Fridman [59] 2008	40/86	15	21	4	39	38	9	0.003	0.9545
Ilhan [60] 2008	78/100	36	32	10	72	26	2	0.038	0.8445
Lin [61] 2008	50/123	19	27	4	73	44	6	0.037	0.8479
Luo [62] 2008	442/195	260	151	31	138	51	6	0.232	0.6298
Soares [63] 2008	12/16	3	9	0	9	5	2	0.825	0.3638
Deshmukh [65] 2009	42/118	22	16	4	52	48	18	1.501	0.2205
Fakhrzadeh [66] 2009	160/76	99	44	17	36	31	9	0.335	0.5628
Ng [67] 2009	38/80	14	14	10	40	32	8	0.181	0.6702
Liu [69] 2011	155/140	58	70	27	74	47	19	5.943	0.0148
Liu H [70] 2011	146/112	54	59	33	61	39	12	2.155	0.1421
Jin [72] 2011	405/400	215	140	50	204	144	52	10.047	0.0015
Ma [73] 2011	122/45	6	115	1	0	44	1	41.172	0
Cao [75] 2012	112/147	33	53	26	49	68	30	0.514	0.4736
Fowdar [76] 2012	377/393	170	174	33	175	183	35	1.746	0.1863
Yin [77] 2012	670/682	244	358	68	322	309	51	3.946	0.0470
Zhang [78] 2012	189/165	128	53	8	117	41	7	1.835	0.1755
Alghasham [79] 2012	26/250	**18**	**8**		**185**	**65**		–	–
Bayramoglu [80] 2013	125/99	65	38	22	56	38	5	0.200	0.6543
Fridman [81] 2013	75/150	29	40	6	71	64	15	0.011	0.9174
Yao [82] 2013	150/150	32	69	49	61	67	22	0.263	0.6078
Yang [83] 2013	200/200	39	99	62	61	89	50	2.303	0.1292
Cai [84] 2014	200/200	39	99	62	61	89	50	2.303	0.1292
Bayramoglu [87] 2015	125/99	65	38	22	56	38	5	0.200	0.6543
Nassereddine [88] 2015	101/102	47	40	14	54	45	3	3.176	0.0747
Wei [90] 2015	246/348	143	82	21	260	78	10	1.888	0.1695
Wen [91] 2015	174/634	45	53	76	258	291	85	0.042	0.8370
Pérez-Razo [92] 2015	372/391	112	174	87	90	200	101	0.222	0.6375
	209/209	34	98	67	35	108	56	1.898	0.1683
Amrani-Midoun [93] 2016	82/72	37	36	9	44	25	3	0.055	0.8142
Fan [94] 2016	214/494	37	102	75	119	234	141	1.272	0.2593
Dwivedi [95] 2017	100/223	71	24	5	184	34	5	4.541	0.0331
Wu [98] 2016	123/120	73	39	11	70	40	10	1.481	0.2235
Ghogomu [99] 2016	41/50	3	24	14	45	5	0	0.139	0.7098
Zhang [100] 2017	220/128	45	122	53	52	56	20	0.569	0.4507
Zhao [101] 2017	200/200	54	99	47	80	91	29	0.143	0.7056
Liu [102] 2019	934/1075	200	439	295	214	505	356	2.060	0.1512
Nong [103] 2019	122/110	15	58	49	35	59	16	1.229	0.2675
Wu [104] 2019	250/200	91	103	56	88	88	24	0.077	0.7816
Zhao [105] 2019	120/120	81	34	5	75	38	7	0.540	0.4623
Candrasatria [106] 2020	213/202	134	73	6	157	42	3	0.010	0.9205

HWE, Hardy–Weinberg equilibrium.

**TABLE 4:** The genotype distribution and HWE of *MTHFR A1298C* polymorphism in this meta-analysis. The bold value is C/T T/T A/C C/C genotype data is incomplete, and HWE evaluation cannot be performed.

First author/Year	Number of samples	Genotype of cases			Genotype of controls			HWE	
	Cases/controls	A/A	A/C	C/C	A/A	A/C	C/C	Chi	*p*
Kahleova [34] 2002	164/173	79	62	23	77	75	21	0.171	0.679
Tylicki [44] 2005	90/90	38	43	9	36	45	9	0.880	0.3481
Markan [52] 2007	153/133	99	43	11	112	17	4	8.277	0.004
Ng [67] 2009	79/39	37	35	7	22	14	3	0.134	0.7143
Wang [107] 2010	195/213	132	56	7	134	68	11	0.377	0.5393
Wang [108] 2010	203/225	138	57	8	139	75	11	0.046	0.8297
Demirel [109] 2011	50/50	25	19	6	14	33	3	7.494	0.0062
Fowdar [76] 2012	368/386	165	151	52	162	173	51	0.201	0.6539
Alghasham [79] 2012	26/250	**15**	**11**		**144**	**106**		–	–
Wei [90] 2015	246/348	157	78	11	213	121	12	1.073	0.3003
Liu [102] 2019	930/1074	679	229	22	801	250	23	0.448	0.5034

HWE, Hardy–Weinberg equilibrium.

### Meta-analysis Results

#### *MTHFR C677T* Polymorphism

In Table 5, we summarized the association between *MTHFR C677T* and EH. In the overall population, EH was found to be at a high risk across all genetic models (TT *vs.* CC: OR = 1.71, 95% CI: 1.44–2.02; TC *vs.* CC: OR = 1.26, 95% CI: 1.15–1.39; TT + TC *vs.* CC: OR = 1.37, 95% CI: 1.24–1.52; TT *vs.* TC + CC: OR = 1.51, 95% CI: 1.31–1.74; T *vs.* C: OR = 1.33, 95% CI: 1.22–1.45).

**TABLE 5:** Pooled results and sensitivity analysis of the association between *MTHFR C677T* polymorphism and essential hypertension. The meaning of bold is in different subgroups, there are statistically significant gene models. In other words, it is the genetic model associated with EH.

Variable	*n* (cases/controls)	TT *vs.* CC		TC *vs.* CC		TT + TC *vs.* CC		TT *vs.* TC + CC		T *vs.* C	
		OR (95%CI)	*P*_h_ / *I* ^2^ (%)	OR (95%CI)	*P*_h_ / *I* ^2^ (%)	OR (95%CI)	*P*_h_ / *I* ^2^ (%)	OR (95%CI)	*P*_h_ / *I* ^2^ (%)	OR (95%CI)	*P*_h_ / *I* ^2^ (%)
Overall	63 (11,556/12,523)	**1.71 (1.44–2.02)** ^a^	<0.001/69.2	**1.26 (1.15–1.39)** ^a^	<0.001/57.0	**1.37 (1.24–1.52)** ^a^	<0.001/66.5	**1.51 (1.31–1.74)** ^a^	<0.001/66.0	**1.33 (1.22–1.45)** ^a^	<0.001/76.6
Ethnicity											
Asian	42 (8,636/9,206)	**1.74 (1.43–2.12)** ^a^	<0.001/71.1	**1.34 (1.21–1.48)** ^a^	0.003/42.0	**1.44 (1.28–1.61)** ^a^	<0.001/63.6	**1.49 (1.26–1.77)** ^a^	<0.001/70.3	**1.35 (1.24–1.46)** ^a^	<0.001/76.0
Caucasian	17 (2,255/2,575)	**1.73 (1.27–2.36)** ^a^	0.012/50.1	1.06 (0.93–1.20)	0.108/31.8	**1.17 (1.04–1.32)**	0.048/39.5	**1.62 (1.34–1.96)**	0.026/45.1	**1.62 (1.34–1.96)** ^a^	0.003/56.6
Mixed	3 (624/692)	0.84 (0.61–1.16)	0.422/0.0	1.06 (0.62–1.79)^a^	0.057/60.2	1.03 (0.64–1.66)^a^	0.078/56.0	1.00 (0.78–1.30)	0.406/0.0	0.99 (0.79–1.25)	0.205/34.6
African	1 (41/50)	**377.00 (18.37–7737.29)**	–	**72.00 (15.83–327.45)**	–	**114.00 (25.56–508.40)**	–	**53.26 (3.06–927.31)**	–	**32.93 (12.05–90.00)**	–
Geographic region											
East Asia	38 (7,924/8,300)	**1.72 (1.40–2.12)** ^a^	<0.001/72.0	**1.31 (1.19–1.45)** ^a^	0.014/36.5	**1.41 (1.26–1.58)** ^a^	<0.001/58.1	**1.47 (1.23–1.76)** ^a^	<0.001/71.7	**1.33 (1.20–1.47)** ^a^	<0.001/75.3
South Asia	2 (253/356)	4.21 (0.84–21.07)	0.239/27.8	**1.60 (1.07–2.40)**	0.549/0.0	**1.82 (1.23–2.67)**	0.767/0.0	3.82 (0.73–20.11)	0.230/30.7	**1.89 (1.34–2.66)**	0.961/0.0
Western Asia	5 (514/624)	2.87 (0.95–8.67)^a^	0.007/75.4	0.97 (0.53–1.79)^a^	0.006/75.7	1.24 (0.72–2.11)^a^	<0.001/74.7	**2.90 (1.14–7.35)** ^a^	0.028/67.0	1.44 (0.83–2.50)^a^	<0.001/84.0
Southeast Asia	2 (459/550)	**3.40 (1.72–6.73)**	0.552/0.0	**1.96 (1.48–2.61)**	0.830/0.0	**2.10 (1.60–2.76)**	0.905/0.0	**2.81 (1.43–5.53)**	0.544/0.0	**1.98 (1.56–2.50)**	0.667/0.0
Europe	5 (727/777)	**1.76 (1.27–2.44)**	0.575/0.0	1.03 (0.82–1.28)	0.440/0.0	1.17 (0.95–1.44)	0.439/0.0	**1.76 (1.30–2.38)**	0.779/0.0	**1.25 (1.08–1.45)**	0.479/0.0
Oceania	3 (714/732)	1.23 (0.86–1.76)	0.380/0.0	1.08 (0.87–1.34)	0.574/0.0	1.10 (0.89–1.36)	0.404/0.0	1.17 (0.83–1.65)	0.549/0.0	1.09 (0.93–1.29)	0.330/9.9
Africa	5 (339/460)	**3.79 (1.02–14.01)** ^a^	0.002/75.8	**2.42 (1.03–5.69)** ^a^	0.000/85.0	**2.78 (1.13–6.83)** ^a^	<0.001/87.5	2.47 (0.84–7.31)^a^	0.015/67.5	**2.32 (1.11–4.84)** ^a^	<0.001/89.6
South America	1 (12/16)	0.54 (0.02–14.35)^a^	–	5.40 (0.98–29.67)^a^	–	3.86 (0.75–19.84)^a^	–	0.23 (0.01–5.30)^a^	–	1.53 (0.50–4.75)^a^	–
North America	2 (614/708)	0.819 (0.53–1.27)	0.219/34.1	0.76 (0.58–1.00)	0.676/0.0	0.77 (0.60–0.99)	0.436/0.0	0.99 (0.70–1.39)	0.235/30.9	0.91 (0.71–1.16)	0.146/48.1
Sensitivity analysis of high-quality and HWE studies											
Overall	20 (4,439/4,661)	**1.14 (1.00–1.30)**	0.032/39.8	1.08 (0.98–1.19)	0.186/21.3	**1.15 (1.01–1.29)** ^a^	0.040/38.1	1.11 (0.99–1.23)	0.159/23.7	**1.14 (1.04–1.25)** ^a^	0.005/50.2
Ethnicity											
Asian	15 (3,067/3,271)	1.17 (0.99–1.36)	0.180/24.8	**1.14 (1.01–1.28)**	0.265/16.8	**1.17 (1.04–1.30)**	0.105/32.9	1.09 (0.96–1.24)	0.333/10.8	**1.10 (1.02–1.19)**	0.018/48.6
Caucasian	4 (800/800)	1.60 (0.87–2.92)^a^	0.067/58.2	1.08 (0.88–1.33)	0.704/0.0	1.14 (0.93–1.39)	0.451/0.0	1.49 (0.86–2.60)^a^	0.083/55.0	1.18 (0.96–1.45)	0.168/40.6
Mixed	1 (572/590)	0.83 (0.60–1.15)^a^	0.113/60.2	0.76 (0.57–1.02)	0.378/0.0	0.78 (0.59–1.02)	0.204/38.0	1.01 (0.78–1.31)^a^	0.151/51.4	0.95 (0.70–1.29)^a^	0.075/68.4
African	–	–	–	–	–	–	–	–	–	–	–
Geographic region											
East Asia	13 (2,701/2,936)	1.13 (0.97–1.32)	0.292/15.1	1.08 (0.95–1.22)	0.721/0.0	1.10 (0.98–1.23)	0.512/0.0	1.07 (0.94–1.22)	0.474/0.0	1.07 (0.99–1.15)	0.261/18.1
South Asia	1 (153/133)	17.00 (0.97–298.31)	–	1.43 (0.82–2.49)	–	**1.71 (1.00–2.94)**	–	15.60 (0.89–272.86)	–	**1.90 (1.17–3.10)**	–
Southeast Asia	1 (213/202)	2.34 (0.58–9.55)	–	**2.04 (1.31–3.18)**	–	**2.06 (1.34–3.17)**	–	1.92 (0.47–7.79)	–	**1.85 (1.26–2.71)**	–
Europe	1 (232/215)	**1.95 (1.04–3.64)**	–	1.32 (0.88–1.96)	–	1.42 (0.97–2.08)	–	1.67 (0.93–3.01)	–	**1.35 (1.03–1.78)**	–
Oceania	2 (467/483)	1.01 (0.64–1.60)	0.755/0.0	0.99 (0.76–1.30)	0.784/0.0	0.99 (0.77–1.29)	0.735/0.0	1.01 (0.65–1.56)	0.811/0.0	1.01 (0.82–1.21)	0.713/0.0
Africa	1 (101/102)	**5.36 (1.45–19.81)**	–	1.02 (0.57–1.82)	–	1.29 (0.75–2.24)	–	**5.31 (1.48–19.10)**	–	1.52 (0.99–2.34)	–
South America	–	–	–	–	–	–	–	–	–	–	–
North America	1 (572/590)	0.83 (0.60–1.15)^a^	0.113/60.2	0.76 (0.57–1.02)	0.378/0.0	0.78 (0.60–1.02)	0.204/38.0	1.01 (0.78–1.31)^a^	0.151/51.4	0.95 (0.70–1.29)^a^	0.075/68.4

a A random-effects model was used.

HWE, Hardy–Weinberg equilibrium.

Next, a subgroup analysis was performed by ethnicity and geographic region. An increased risk of hypertension could be found in Asian (TT *vs.* CC: OR = 1.74, 95% CI: 1.43–2.12; TC *vs.* CC: OR = 1.34, 95% CI: 1.21–1.48; TT + TC *vs.* CC: OR = 1.44, 95% CI: 1.28–1.61; TT *vs.* TC + CC: OR = 1.49, 95% CI: 1.26–1.77; T *vs.* C: OR = 1.35, 95% CI: 1.24–1.46), Caucasian (TT *vs.* CC: OR = 1.73, 95% CI: 1.27–2.36; TT + TC *vs.* CC: OR = 1.17, 95% CI: 1.04–1.32; TT *vs.* TC + CC: OR = 1.62, 95% CI: 1.34–1.96; T *vs.* C: OR = 1.62, 95% CI: 1.34–1.96), and African (TT *vs.* CC: OR = 377.00, 95% CI: 18.37–7737.29; TC *vs.* CC: OR = 72.00, 95% CI: 15.83–327.45; TT + TC *vs.* CC: OR = 114.00, 95% CI: 25.56–508.40; TT *vs.* TC + CC: OR = 53.26, 95% CI: 3.06–927.31; T *vs.* C: OR = 32.93, 95% CI: 12.05–90.00). The same happened in the East Asia region (TT *vs.* CC: OR = 1.72, 95% CI: 1.40–2.12; TC *vs.* CC: OR = 1.31, 95% CI: 1.19–1.45; TT + TC *vs.* CC: OR = 1.41, 95% CI: 1.26–1.58; TT *vs.* TC + CC: OR = 1.47, 95% CI: 1.23–1.76; T *vs.* C: OR = 1.33, 95% CI: 1.20–1.47), South Asia region (TC *vs.* CC: OR = 1.60, 95% CI: 1.07–2.40; TT *vs.* TC + CC: OR = 1.82, 95% CI: 1.23–2.67; T *vs.* C: OR = 1.89, 95% CI: 1.34–2.66), Southeast Asia region (TT *vs.* CC: OR = 3.40, 95% CI: 1.72–6.73; TC *vs.* CC: OR = 1.96, 95% CI: 1.48–2.61; TT + TC *vs.* CC: OR = 2.10, 95% CI: 1.60–2.76; TT *vs.* TC + CC: OR = 2.81, 95% CI: 1.43–5.53; T *vs.* C: OR = 1.98, 95% CI: 1.56–2.50), Europe region (TT *vs.* CC: OR = 1.76, 95% CI: 1.27–2.44; TT *vs.* TC + CC: OR = 1.76, 95% CI: 1.30–2.28; T *vs.* C: OR = 1.25, 95% CI: 1.08–1.45), and Africa region (TT *vs.* CC: OR = 3.79, 95% CI: 1.02–14.01; TC *vs.* CC: OR = 2.42, 95% CI: 1.03–5.69; TT + TC *vs.* CC: OR = 2.78, 95% CI: 1.13–6.83; T *vs.* C: OR = 2.32, 95% CI: 1.11–4.84).

#### *MTHFR A1298C* Polymorphism

The association between *MTHFR A1298C* and EH is shown in Table 6. No significant association was found in both the overall population and the subgroup analyzed by ethnicity. However, according to the subgroup analyzed by geographical origin, a clear risk correlation between the two was found in South Asia region (AC *vs.* AA: OR = 2.86, 95% CI: 1.53–5.34; CC + AC *vs.* AA: OR = 2.91, 95% CI: 1.64–5.15; C *vs.* A: OR = 2.60, 95% CI: 1.59–4.26). A conservation correlation has been found in the West Asia region (CC + AC *vs.* AA: OR = 0.39, 95% CI: 0.17–0.89).

**TABLE 6:** Pooled results and sensitivity analysis of the association between *MTHFR A1298C* polymorphism and essential hypertension. The meaning of bold is in different subgroups, there are statistically significant gene models. In other words, it is the genetic model associated with EH.

Variable	*n* (cases/controls)	CC *vs.* AA		AC *vs.* AA		CC + AC *vs.* AA		CC *vs.* .AC + AA		C *vs.* A	
		OR (95%CI)	*P*_h_ / *I* ^2^ (%)	OR (95%CI)	*P*_h_ / *I* ^2^ (%)	OR (95%CI)	*P*_h_ / *I* ^2^ (%)	OR (95%CI)	*P*_h_ / *I* ^2^ (%)	OR (95%CI)	*P*_h_ / *I* ^2^ (%)
Overall	11 (2,504/2,979)	1.07 (0.84–1.37)	0.814/0.0	0.94 (0.77–1.17)^a^	0.010/56.9	0.98 (0.87–1.10)^a^	0.004/62.7	1.12 (0.89–1.43)	0.889/0.0	1.01 (0.86–1.18)^a^	0.015/56.0
Ethnicity											
Caucasian	6 (777/988)	1.03 (0.74–1.44)	0.994/0.0	0.84 (0.64–1.10)	0.230/27.3	0.87 (0.71–1.07)	0.245/26.4	1.14 (0.83–1.55)	0.929/0.0	0.96 (0.82–1.11)	0.724/0.0
Asian	5 (1,727/1,991)	1.11 (0.77–1.60)	0.292/19.2	1.05 (0.77–1.44)^a^	0.007/71.9	1.038 (0.90–1.20)^a^	0.002/76.5	1.11 (0.77–1.60)	0.488/0.0	1.07 (0.80–1.43)^a^	0.001/77.5
Geographic region											
Europe	2 (243/212)	1.12 (0.61–2.05)	0.748/0.0	0.99 (0.56–1.76)	0.199/39.3	0.98 (0.67–1.42)	0.238/28.3	1.18 (0.66–2.10)	0.988/0.0	1.03 (0.77–1.36)	0.385/0.0
Oceania	2 (458/476)	0.99 (0.66–1.49)	0.923/0.0	0.87 (0.66–1.14)	0.876/0.0	0.89 (0.69–1.160)	0.941/0.0	1.07 (0.73–1.57)	0.885/0.0	0.96 (0.79–1.16)	0.979/0.0
South Asia	1 (153/113)	3.11 (0.96–10.08)	–	**2.86 (1.53–5.34)**	–	**2.91 (1.64–5.15)**	–	2.50 (0.78–8.04)	–	**2.60 (1.59–4.26)**	–
East Asia	3 (1,328/1,512)	0.91 (0.58–1.42)	0.554/0.0	0.95 (0.76–1.18)	0.254/27.0	0.98 (0.83–1.15)	0.186/40.6	0.93 (0.60–1.44)	0.662/0.0	0.93 (0.75–1.15)	0.171/43.4
Western Asia	2 (76/300)	1.12 (0.24–5.19)	–	0.57 (0.19–1.73)	0.063/71.1	**0.39 (0.17–0.89)**	–	2.14 (0.50–9.07)	–	0.70 (0.39–1.26)	–
Southeast Asia	1 (246/346)	1.24 (0.54–2.89)	–	0.88 (0.62–1.24)^a^	–	0.91 (0.65–1.27)^a^	–	1.30 (0.57–3.00)	–	0.96 (0.72–1.28)^a^	–
Sensitivity analysis of high-quality and HWE studies											
Overall	5 (1,786/1,988)	0.95 (0.71–1.29)	0.867/0.0	0.95 (0.82–1.09)	0.506/0.0	0.95 (0.83–1.09)	0.451/0.0	1.01 (0.75–1.34)	0.900/0.0	0.97 (0.86–1.08)	0.470/0.0
Ethnicity											
Caucasian	2 (458/476)	0.99 (0.66–1.49)	0.923/0.0	0.87 (0.66–1.14)	0.876/0.0	0.89 (0.69–1.16)	0.941/0.0	1.07 (0.73–1.57)	0.885/0.0	0.96 (0.79–1.16)	0.979/0.0
Asian	3 (1,328/1,512)	0.91 (0.58–1.42)	0.554/0.0	0.98 (0.83–1.16)	0.254/27.0	0.98 (0.83–1.15)	0.186/40.6	0.928 (0.60–1.44)	0.662/0.0	0.97 (0.84–1.12)	0.171/43.4
Geographic region											
Europe	–	–	–	–	–	–	–	–	–	–	–
Oceania	2 (458/476)	0.99 (0.66–1.49)	0.923/0.0	0.87 (0.66–1.14)	0.876/0.0	0.89 (0.69–1.16)	0.941/0.0	1.07 (0.73–1.57)	0.885/0.0	0.96 (0.79–1.16)	0.979/0.0
East Asia	3 (1,328/1,512)	0.91 (0.58–1.42)	0.554/0.0	0.98 (0.83–1.16)	0.254/27.0	0.98 (0.83–1.15)	0.186/40.6	0.93 (0.60–1.44)	0.662/0.0	0.97 (0.84–1.12)	0.171/43.4

a A random-effects model was used.

HWE, Hardy–Weinberg equilibrium.

### Heterogeneity and Sensitivity Analyses

In Table 5, we showed the very obvious heterogeneity. A meta-regression analysis was performed based on geographic origin, ethnicity, control group origin, gene quality control, matching, sample size, HWE, and literature quality assessment. Ethnicity was the source of heterogeneity in the final results (TC *vs.* CC: *P* = 0.035; TT + TC *vs.* CC: *P* = 0.042).

A sensitivity analysis was performed to rule out individual studies one by one, and the results were found to be stable. When we restricted the high-quality and HWE studies, there was no significant change in *A1298C* genotype. However, the results of the *C677T* genotype changed significantly in the overall population (TC *vs.* CC: OR = 1.08, 95% CI: 0.98–1.19, *P*_h_ = 0.186, *I* ^2^ = 21.3%; TT *vs.* TC + CC: OR = 1.11, 95% CI: 0.99–1.23, *P*_h_ = 0.159, *I* ^2^ = 23.7%), Asian population (TT *vs.* CC: OR = 1.17, 95% CI: 0.99–1.36, *P*_h_ = 0.180, *I* ^2^ = 24.8%; TT *vs.* TC + CC: OR = 1.09, 95% CI: 0.96–1.24, *P*_h_ = 0.333, *I* ^2^ = 10.8%), Caucasian population (TT *vs.* CC: OR = 1.60, 95% CI: 0.87–2.92, *P*_h_ = 0.067, *I* ^2^ = 58.2%; TT + TC *vs.* CC: OR = 1.14, 95% CI: 0.93–1.39, *P*_h_ = 0.451, *I* ^2^ = 0.0%; TT *vs.* TC + CC: OR = 1.49, 95% CI: 0.86–2.60, *P*_h_ = 0.083, *I* ^2^ = 55.0%; T *vs.* C: OR = 1.18, 95% CI: 0.96–1.45, *P*_h_ = 0.168, *I* ^2^ = 40.6%), and East Asia region (TT *vs.* CC: OR = 1.13, 95% CI: 0.97–1.32, P_h_ = 0.292, *I* ^2^ = 15.1%; TC *vs.* CC: OR = 1.08, 95% CI: 0.95–1.22, *P*_h_ = 0.720, *I* ^2^ = 0.0%; TT + TC *vs.* CC: OR = 1.10, 95% CI: 0.98–1.23, *P*_h_ = 0.512, *I* ^2^ = 0.0%; TT *vs.* TC + CC: OR = 1.07, 95% CI: 0.94–1.22, *P*_h_ = 0.474, *I* ^2^ = 0.0%; T *vs.* C: OR = 1.07, 95% CI: 0.99–1.15, *P*_h_ = 0.261, *I* ^2^ = 18.1%). All results are shown in Table 5 and Table 6.

### Publication Bias

**FIGURE 2:**
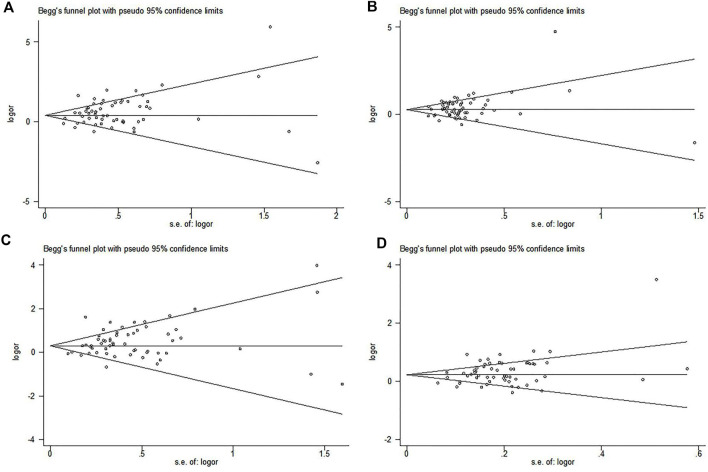
Begg’s funnel plot to assess the publication bias of *MTHFR* *C677T* polymorphism in the overall population. **(A)** Allelic model, T *vs.* C; **(B)** dominant model, TT + TC *vs.* CC; **(C)** recessive model, TT *vs.* CC + TC; **(D)** homozygote genetic model, TT *vs.* CC.

**FIGURE 3:**
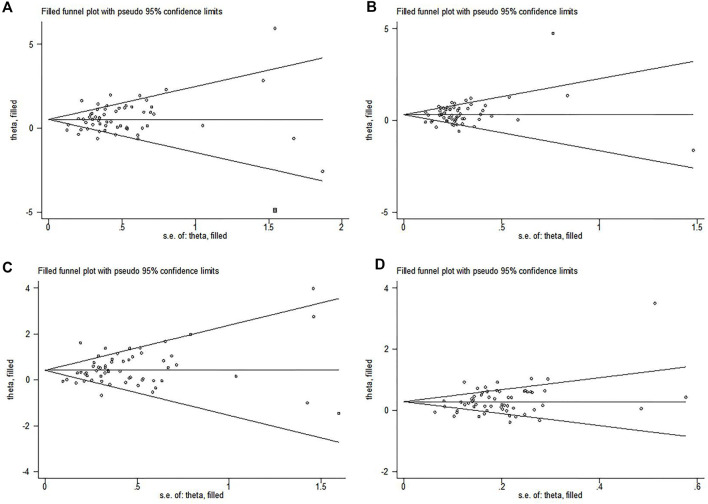
Trim-and-fill plots of the publication bias to assess *MTHFR C677T* polymorphism in the overall population. **(A)** Allelic model, T *vs.* C; **(B)** dominant model, TT + TC *vs.* CC; **(C)** recessive model, TT *vs.* CC + TC; **(D)** homozygote genetic model, TT *vs.* CC.

Begg’s funnel plot and Egger test were used to evaluate publication bias. For *MTHFR C677T* polymorphism, there was publication bias in four genetic models (TT *vs.* CC: *P* = 0.009; TT + TC *vs.* CC: *P* = 0.045; TT *vs.* TC + CC: *P* = 0.007; T *vs.* C: *P* = 0.005) (Figure 2). In order to further clarify the publication bias, we applied the trim-and-fill method, and the results of the overall population did not change (Figure 3). For *MTHFR A1298C* polymorphism, the shape of the funnel plot was uniform and symmetrical, indicating that there was no significant publication bias under all genetic models (*P*).

### Cumulative Meta-analysis Results

**FIGURE 4:**
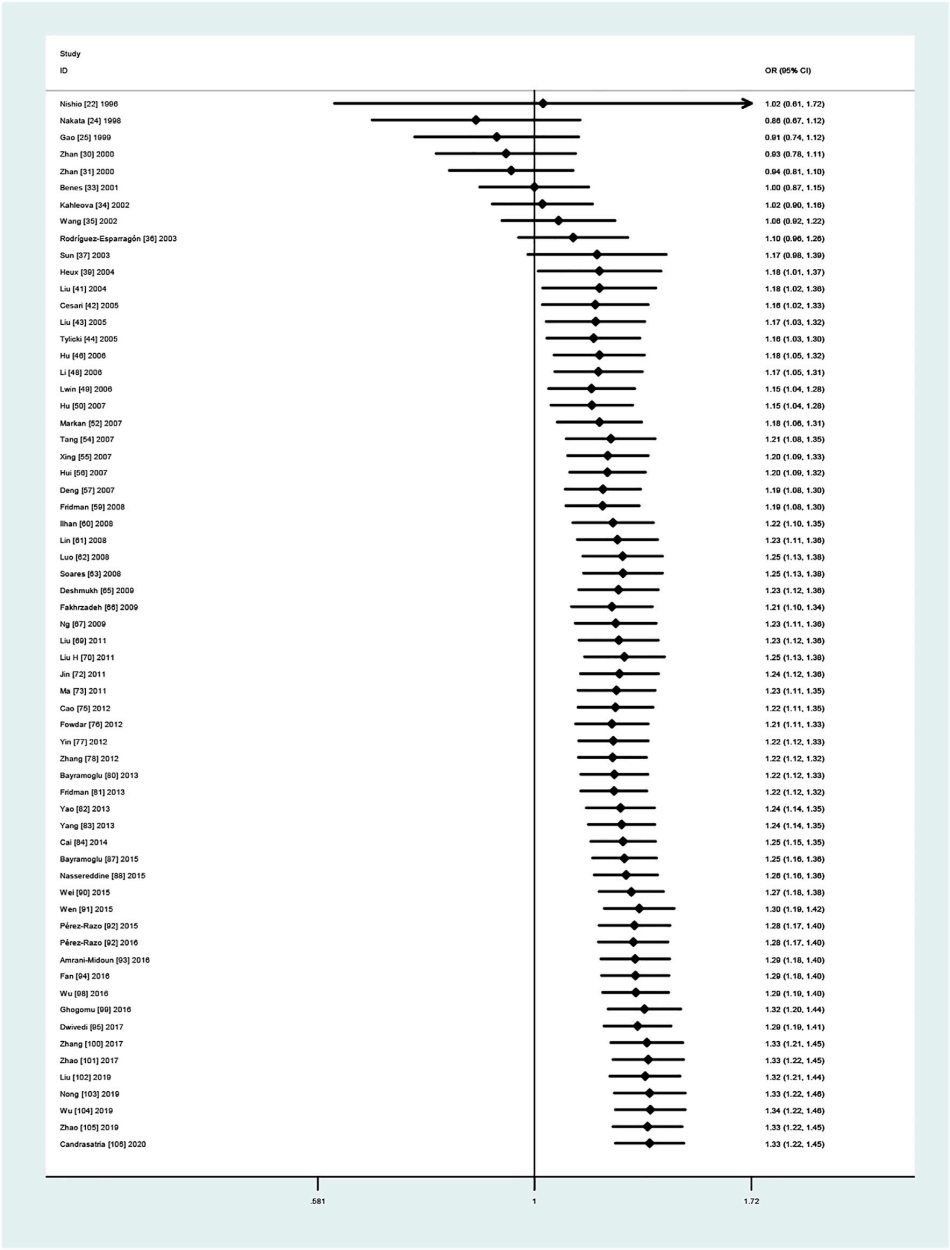
A cumulative meta-analysis of forest plots was performed based on years (allelic model, T *vs.* C).

According to year, the literature was included sequentially to evaluate the stability of cumulative effect size. The results showed that the correlation degree tended to be stable (Figure 4).

### Credibility of the Positive Results

After FPRP, BFDP, and Venice criterion evaluation, results that met both FPRP <0.2, BFDP <0.8, and above moderate epidemiological evidence were only in the heterozygote (TC *vs.* CC) and recessive model (TT *vs.* CT + CC). Table 7 shows the details. This was different from the results of the sensitivity analyses that limited high-quality and HWE studies.

**TABLE 7:** The credibility results of the positive effects after FPRP, BFDP, and Venice criterion.

Variables	Model	OR (95% CI)	*I* ^2^ (%)	Credibility		
				Prior probability of 0.001		Venice criteria
				FPRP	BFDP	
*MTHFR C677T*						
Overall	TT *vs.* CC	1.71 (1.44–2.02)	69.2	<0.001	<0.001	ACC
Ethnicity						
Asian	TT *vs.* CC	1.74 (1.43–2.12)	71.1	0.001	0.002	ACB
Caucasian	TT *vs.* CC	1.73 (1.27–2.36)	50.1	0.746	0.935	ACB
Geographic region						
East Asia	TT *vs.* CC	1.72 (1.40–2.12)	72.0	0.004	0.020	ACC
Europe	TT *vs.* CC	1.76 (1.27–2.44)	0.0	0.804	0.942	AAB
Africa	TT *vs.* CC	3.79 (1.02–14.01)	75.8	0.998	0.998	BCC
Sensitivity analysis (only studies with high quality and HWE)						
Overall	TT *vs.* CC	1.14 (1.00–1.30)	39.8	0.981	0.999	ABC
Overall	TC *vs.* CC	1.26 (1.15–1.39)	57.0	0.004	0.226	ACB
Ethnicity						
Asian	TC *vs.* CC	1.34 (1.21–1.48)	42.0	<0.001	0.001	ABB
Geographic region						
East Asia	TC *vs.* CC	1.31 (1.19–1.45)	36.5	<0.001	0.015	ABC
Africa	TC *vs.* CC	2.42 (1.03–5.69)	85.0	0.997	0.998	ACC
Sensitivity analysis (only studies with high quality and HWE)						
Ethnicity						
Asian	TC *vs.* CC	1.14 (1.01–1.28)	16.8	0.964	0.999	AAB
Overall	TT + TC *vs.* CC	1.37 (1.24–1.52)	66.5	<0.001	<0.001	ACC
Ethnicity						
Asian	TT + TC *vs.* CC	1.44 (1.28–1.61)	63.6	<0.001	<0.001	ACB
Caucasian	TT + TC *vs.* CC	1.17 (1.04–1.32)	39.5	0.915	0.997	ABB
Geographic region						
East Asia	TT + TC *vs.* CC	1.41 (1.26–1.58)	58.1	<0.001	<0.001	ACC
Africa	TT + TC *vs.* CC	2.78 (1.13–6.83)	87.5	0.997	0.997	ACC
Sensitivity analysis (only studies with high quality and HWE)						
Overall	TT + TC *vs.* CC	1.15 (1.01–1.29)	38.1	0.945	0.998	ABB
Ethnicity						
Asian	TT + TC *vs.* CC	1.17 (1.04–1.30)	32.9	0.777	0.993	ABC
Overall	TT *vs.* TC + CC	1.51 (1.31–1.74)	66.0	<0.001	0.001	ACC
Ethnicity						
Asian	TT *vs.* TC + CC	1.49 (1.26–1.77)	70.3	0.011	0.217	ACB
Caucasian	TT *vs.* TC + CC	1.62 (1.34–1.96)	45.1	0.003	0.036	ABB
Geographic region						
East Asia	TT *vs.* TC + CC	1.47 (1.23–1.76)	71.7	0.045	0.541	ACB
Europe	TT *vs.* TC + CC	1.76 (1.30–2.38)	0.0	0.617	0.887	AAB
Overall	T *vs.* C	1.33 (1.22–1.45)	76.6	<0.001	<0.001	ACC
Ethnicity						
Asian	T *vs.* C	1.35 (1.24–1.46)	76.0	<0.001	<0.001	ACB
Caucasian	T *vs.* C	1.62 (1.34–1.96)	56.6	0.003	0.036	ACB
Geographic region						
East Asia	T *vs.* C	1.33 (1.20–1.47)	75.3	<0.001	0.002	ACC
Europe	T *vs.* C	1.25 (1.08–1.45)	0.0	0.764	0.991	AAB
Africa	T *vs.* C	2.32 (1.11–4.84)	89.6	0.995	0.997	BCC
Sensitivity analysis (only studies with high quality and HWE)						
Overall	T *vs.* C	1.14 (1.04–1.25)	50.2	0.841	0.996	ACC
Ethnicity						
Asian	T *vs.* C	1.10 (1.02–1.19)	48.6	0.946	0.999	ABC
*MTHFR A1298C*						
Geographic region						
Western Asia	CC + AC *vs.* AA	0.39 (0.17–0.89)	–	0.996	0.997	B––

FPRP, false-positive report probability; BFDP, Bayesian false discovery probability; HWE, Hardy–Weinberg equilibrium.

## Discussion

In 1996, a group of researchers first investigated the association between *MTHFR C677T* polymorphism and EH in Japan (Nishio et al., 1996). Then, another group of researchers studied *MTHFR C677T* and *A1298C* polymorphisms for the first time in the Czech Republic (Kahleová et al., 2002). Thereafter, many scholars worldwide partook in the research, but the results were inconsistent and controversial. Even though several meta-analyses (Qian et al., 2007; Niu et al., 2012; Yang B. et al., 2014; Yang K.-M. et al., 2014; Wu et al., 2014; Fu et al., 2019) appeared to investigate the association between the *MTHFR* SNPs, methodological problems, especially the inclusion of pregnancy-related hypertension and lack of quality assessment of the studies included, hampered the acceptance of their findings. Therefore, it is necessary to conduct a new, more reliable, and more comprehensive meta-analysis.

After data collection for this meta-analysis, HWE compliance checks of the control group, and literature quality score assignments, the effect sizes were combined using five genetic models. Ultimately, the *C677T* polymorphism was found to be a risk genotype in the overall population, East Asians, Southeast Asians, South Asians, Caucasians/Europeans (allelic model, homozygote model, dominant model, and recessive model), and Africans. It was possible that both were linked in the study population from African, South Asia region, and Southeast Asia region (Table 5). However, we are of the opinion that, based on a lack of studies on populations representative of Africa [one report (Ghogomu et al., 2016)], South Asia [two reports (Markan et al., 2007; Dwivedi and Sinha, 2017)], and South-East Asia [two reports (Wei et al., 2015; Candrasatria et al., 2020)], these results should be interpreted with caution until more studies on these underrepresented groups accumulate (Higgins, 2008). Then, we conducted a sensitivity analysis to choose high-quality studies and studies whose populations adhered to the assumptions of HWE. The results showed that *C677T* polymorphism was significantly associated with EH only in the overall population (allele, dominant, and homozygote model) and the Asian population (dominant, allele, and heterozygote model). When we used the FPRP, BFDP test, and the Venice criterion for evaluation, all significant associations were less-credible positive results, indicating that the association could not be noteworthy and could therefore be regarded as weak-level evidence. These tests are necessary to avoid random error and confounding bias, but sometimes they can alter the results of the epidemiological studies (Wakefield, 2007; Ioannidis et al., 2008; Zhao et al., 2018; Tian, 2019). At the same time, among the low-quality studies, there were four HWD studies, 28 unadjusted studies, 34 gene quality uncontrolled studies, and 34 no matching studies, which may be the main source of bias. In addition, we did not find evidence that the *A1298C* polymorphism was associated with EH. The South Asia [one report (Markan et al., 2007)] and Southeast Asia [one report (Wei et al., 2015)] results were also significantly less reliable because of the small number of studies (Tables 5–7). In conclusion, our findings are comparable to previous reports which observed that the *MTHFR* variant SNP at position *677* conveys a risk for hypertension; however, compared with the previous meta-analyses, the FPRP, BFDP, and Venice criterion indicated that this relationship is probably not causal. Some observational studies have shown that genetic changes are inseparable from the impact of environmental factors such as diet, exercise, smoking, and drinking. A method to explore the connection between genes and environmental factors is to study people in different regions and cultures (Anand, 2005). Hcy is an independent risk for EH (Zhong et al., 2017). Alam *et al*. found that the *MTHFR 677 TT* genotype was associated with hHcy in Indians (Alam et al., 2008). However, no association between *MTHFR C677T* polymorphism and hHcy was found in long-term resident Indians in the UK (Chambers et al., 2000). Further research suggested that the difference may be caused by different environmental factors such as diet and exercise.

We could find a significant heterogeneity between the articles included in the final results (Table 5 and Table 6). Heterogeneity exists naturally in meta-analysis, so sources of heterogeneity should be sought as far as possible. First, after excluding each study one by one, we believe that the included studies are within the confidence interval of the total effect size. We performed a sensitivity analysis which, in certain instances, can be useful to identify the source of heterogeneity. Therefore, we conducted a meta-regression analysis and found that ethnicity was the source of heterogeneity only in the dominant model and the heterozygote model. In addition, publication bias was also observed (Figure 2 suggests that small sample studies lead to publication bias). In order to identify and correct publication bias caused by the asymmetry of a funnel figure (Rothstein et al., 2005), we applied trim-and-fill method to remove the small sample study and (to) include missing studies. The results showed that there were no obvious changes before and after the cut to fill the consolidation effect (before the cut to fill the consolidation effect was statistically significant, while after the cut to fill the consolidation effect was statistically significant) (Figure 3); publication bias may not exist (Zhang and Zhong, 2009). In molecular epidemiological studies, small-sample research is very likely to have random errors and bias, resulting in unreliable final results (Ioannidis et al., 2008). Because the experimental design of small-sample research is not strict, its research quality is often low. Cumulative meta-analysis was carried out according to year. The results showed that, with the extension of year, the effect size did not change much, and the final results were gradually stable (Wang et al., 2009) (Figure 4). When we carried out a strict and high-quality design for small-sample research, the results were close to the real level.

Compared with previously published meta-analyses, this study has several advantages. Firstly, we have the largest sample size to date. A total of 66 case–control studies were included. For *MTHFR C677T* polymorphism, 63 studies were collected, including 11,556 case groups and 12,523 control groups. For *MTHFR A1298C* polymorphism, there were 11 studies with 2,504 case groups and 2,979 control groups. Secondly, we analyzed the sensitivity of the combined results by excluding individual studies one by one and limiting high-quality and HWE studies. Thirdly, meta-regression, cumulative meta-analysis, trim-and-fill method, and geographic origin subgroup analysis were used to verify the qualified combined effect size. Fourthly, we used the FPRP and BFDP tests that were first introduced to judge the credibility of the positive results. The Venice criterion was used to assess the cumulative level of epidemiological evidence for genetic association. The GWAS on Europeans was an association study for the detection of multiple SNPs. The method was regression analysis. An assumption of regression analysis is the independent distribution of data. However, the fact is that many individuals may have distant relationships, which will lead to false-positive results in an association analysis (O'Donnell and Nabel, 2008; Sul et al., 2018). However, our study conducted a credibility analysis of positive results obtained by traditional methods.

However, there are also shortcomings to our meta-analysis that should be kept in mind. Firstly, only Chinese and English studies were included in this study; studies in other languages were not included. There is a certain degree of heterogeneity. Secondly, this study did not include the data of family genetic aggregation, smoking, body weight, body mass index, gender, age, and medication history. Therefore, the results may be affected by confounding factors. Thirdly, the sources of the control group were not uniform, which may have classification bias. Fourthly, there were few studies on *MTHFR C677T* polymorphism in Africa, South Asia, Southeast Asia, and West Asia. Therefore, heterogeneity may exist. More research should be done in these areas. Fifthly, with regard to the *MTHFR A1298C* polymorphism, since there were only two studies in West Asia, it is still uncertain whether this SNP is associated with EH.

## Conclusion

In summary, this study provided a comprehensive analysis of *MTHFR* polymorphisms on the risk of EH in different populations worldwide. The results showed that *MTHFR C677T* gene polymorphism was associated with increased EH risks in overall population, East Asian, and Caucasian. However, after FPRP, BFDP, and Venice Criteria tests, the above-mentioned associations became less reliable. These results should be treated with caution because they could be false-positives. The final conclusion highlighted weak epidemiological credibility for a higher risk of EH of the *MTHFR 677T* allele. Therefore, more investigations are needed to provide researchers with conclusive evidence of whether *MTHFR C677T* is a genetic predictor of EH. Similarly, there was no significant association between the *MTHFR A1298C* polymorphism and EH. Therefore, *MTHFR* polymorphisms could not contribute to the development of hypertension. In the future, more studies are needed to repeatedly verify the current conclusions.

## Data Availability

The raw data supporting the conclusions of this article will be made available by the authors, without undue reservation.

## Abbreviations

BFDP, Bayesian false discovery probability; CI, confidence intervals; CNKI, China National Knowledge Infrastructure; EH, essential hypertension; FPRP, false-positive report probability; MTHFR, 5,10-methylenetetrahydrofolate reductase; OR, odds ratios; SNP, single-nucleotide polymorphism.
